# Intraductal Papillary Neoplasm of Bile Duct: Updated Clinicopathological Characteristics and Molecular and Genetic Alterations

**DOI:** 10.3390/jcm9123991

**Published:** 2020-12-09

**Authors:** Yasuni Nakanuma, Katsuhiko Uesaka, Yuko Kakuda, Takashi Sugino, Keiichi Kubota, Toru Furukawa, Yuki Fukumura, Hiroyuki Isayama, Takuro Terada

**Affiliations:** 1 Shizuoka Cancer Center, Department of Diagnostic Pathology, Shizuoka 411-8777, Japan; y.kakuda@scchr.jp (Y.K.); t.sugino@scchr.jp (T.S.); 2 Department of Diagnostic Pathology, Fukui Prefecture Saiseikai Hospital, Fukui 918-8503, Japan; 3 Shizuoka Cancer Center, Department of Hepatobiliary Pancreatic Surgery, Shizuoka 411-8777, Japan; k.uesaka@scchr.jp; 4 Department of Hepatobiliary Pancreatic Surgery, Dokkyo University Hospital, Tochigi 321-0293, Japan; kubotak@dokkyomed.ac.jp; 5 Department of Investigative Pathology, Tohoku University Graduate School of Medicine, Sendai 980-8574, Japan; toru.furukawa@med.tohoku.ac.jp; 6 Deparatment of Human Pathology, Juntendo University School of Medicine, Tokyo 113-8431, Japan; yfuku@juntendo.ac.jp; 7 Department of Endoscopy, Juntendo University School of Medicine, Tokyo 113-8431, Japan; h-isayama@juntendo.ac.jp; 8 Department of Gastrointestinal Surgery, Fukui Prefecture Saiseikai Hospital, Fukui 918-8503, Japan; terada.takudo@fukui.saiseikai.or.jp

**Keywords:** cholangiocarcinoma, preinvasive lesion, intraductal papillary neoplasm of bile duct, biliary tree, intraductal papillary mucinous neoplasm of pancreas

## Abstract

Intraductal papillary neoplasm of the bile duct (IPNB), a pre-invasive neoplasm of the bile duct, is being established pathologically as a precursor lesion of invasive cholangiocarcinoma (CCA), and at the time of surgical resection, approximately half of IPNBs show stromal invasion (IPNB associated with invasive carcinoma). IPNB can involve any part of the biliary tree. IPNB shows grossly visible, exophytic growth in a dilated bile duct lumen, with histologically villous/papillary neoplastic epithelia with tubular components covering fine fibrovascular stalks. Interestingly, IPNB can be classified into four subtypes (intestinal, gastric, pancreatobiliary and oncocytic), similar to intraductal papillary mucinous neoplasm of the pancreas (IPMN). IPNBs are classified into low-grade and high-grade based on lining epithelial features. The new subclassification of IPNB into types 1 (low-grade dysplasia and high-grade dysplasia with regular architecture) and 2 (high-grade dysplasia with irregular architecture) proposed by the Japan–Korea pathologist group may be useful in the clinical field. The outcome of post-operative IPNBs is more favorable in type 1 than type 2. Recent genetic studies using next-generation sequencing have demonstrated the existence of several groups of mutations of genes: (i) IPNB showing mutations in *KRAS*, *GNAS* and *RNF43* belonged to type 1, particularly the intestinal subtype, similar to the mutation patterns of IPMN; (ii) IPNB showing mutations in *CTNNB1* and lacking mutations in *KRAS*, *GNAS* and *RNF43* belonged to the pancreatobiliary subtype but differed from IPMN. IPNB showing mutation of *TP53*, *SMAD4* and *PIK3CA* might reflect complicated and other features characterizing type 2. The recent recognition of IPNBs may facilitate further clinical and basic studies of CCA with respect to the pre-invasive and early invasive stages.

## 1. Introduction

The concept of epithelial tumors arising from non-invasive intraepithelial dysplasia or neoplasm is well-established in various human cancers [1]. Recent studies have shown that there are at least two types of pre-invasive neoplasms of the bile ducts preceding cholangiocarcinoma (CCA): biliary intraepithelial neoplasm (BilIN) and intraductal papillary neoplasm of the bile duct (IPNB) [2,3,4,5,6,7,8,9]. BilINs are microscopically identifiable intraepithelial epithelial neoplasms and may be the most common precursor of nodular sclerosing, perihilar and distal CCA (p/dCCA) and large-duct intrahepatic CCA (iCCA) [4,5,6,7,10,11,12,13,14]. In contrast, IPNB has unique clinicopathological features and is defined as an intraductal growing tumor, developing in the intrahepatic and extrahepatic bile ducts [2,3,9,15,16,17,18]. About half of IPNBs show stromal invasion at the time of surgical resection. Mucinous cystic neoplasm (MCN) is another pre-invasive intraepithelial neoplasm associated with ovarian-like stroma and lacks communication with the bile duct lumen [19,20].

Historically, IPNBs have been studied with reference to intraductal papillary mucinous neoplasm of the pancreas (IPMN), as the biliary tree and pancreas are located closely anatomically, and at least some biliary diseases show similarities to pancreatic diseases [2,21,22,23,24,25,26]. Through these comparative studies, the main pathological characteristics of IPNB have been recognized, including the presence of four subtypes, slow progression with intraepithelial mucosal spreading around the main tumor and mucus hypersecretion. The radiological comparison of biliary diseases, including IPNB, with their pancreatic counterparts has also been attempted [27,28,29]. Approximately half of IPNBs reportedly showed histopathological features similar to those of IPMNs [30,31,32,33]. However, IPNB differed from IPMN in its higher histological grade, more advanced stage, higher frequency of associated invasive cancer, worse prognosis and some differences in the oncogenic signal pathways and genetic changes [24,25,26,34]. According to recent studies including such comparative processes, IPNB is now being established as an independent disease along the biliary tree. While IPNBs have been given several different names reflecting their characteristic features, the World Health Organization (WHO) published the Classification of Digestive System Tumours 5th edition (2019), in which the only term IPNB was proposed using one chapter (Table 1) [3].

We herein review the pathological features of IPNB, based on this WHO classification [3], with reference to the clinical and molecular/genetic features and long-term post-operative outcomes.

## 2. Clinical Features, Epidemiology and Imaging and Endoscopic Findings of IPNBs

IPNB is a recently defined pathologic entity [2,3,35] and premalignant disease characterized by a low incidence, high risk of malignant transformation and an uncertain prognosis [36]. Its clinical characteristics and classification as well as radiological features have yet to be established [15,31,35].

### 2.1. Clinical Features, Epidemiology and Risks, Related Diseases and Complication

#### 2.1.1. Clinical Features

IPNBs typically affect middle-aged to elderly adults and show a slight male predominance [37,38,39,40,41]. Intermittent or recurrent, right-upper-quadrant abdominal pain, fever and acute cholangitis or jaundice are the most common clinical manifestations, but a certain percentage of patients (about 12%) have no symptoms at the diagnosis [15,35,39]. Elevated levels of alkaline phosphatase, carcinoembryonic antigen (CEA) and carbohydrate antigen 19-9 (CA19-9) have been reported, although they are unlikely to have high sensitivity or specificity for the diagnosis of IPNB. The serum levels of CA19-9 may reflect the tumor burden and level of invasiveness [36,41]. Notably, the clinicopathological features, prognosis and surgical methods differ between IPNB of the intrahepatic and extrahepatic bile ducts (see below) [15,35,37].

#### 2.1.2. Epidemiology and Risks

IPNB is a rare disease entity with a prevalence of 4% to 15% among bile duct tumors [35,37,42]. IPNB was mainly reported in East Asia, and the incidence is regarded to be higher in these countries than in others [15,39,40,43,44]. Zen et al. [45] examined the ratio of IPNB/mucinous cystic neoplasm of liver (MCN-L) and showed this ratio to be 5.7:1 in Seoul but 1:3.0 in Seattle (WA, USA) and 1:6.3 in London (UK). This difference was mainly attributable to the considerably greater number of IPNB patients in Seoul than in Seattle and London. Hepatolithiasis and liver fluke infection (*Clonorchiasis sinensis* (CS) or *Opisthorchis viverrini* (OV) infection) are major risk factors of IPNB in East Asian countries [46,47,48,49]. Furthermore, patients with IPNB are frequently accompanied by cholecystolithiasis and choledocholithiasis [15]. IPNB identified in centers from Asia was more likely to be intrahepatic and less frequently invasive than those cases in Western centers [35,37,40]. IPNBs also reportedly develop in primary sclerosing cholangitis (PSC) and congenital biliary tract disease [15,50,51]. Interestingly, these etiologic factors are also known as major risk factors for nodular-sclerosing p/dCCA, large-duct iCCA and BilIN, suggesting that these factors may be causally related to the development of IPNB and also of conventional CCA, probably via the BilIN process [2,3,6,52].

Recently, an outbreak of IPNB was reported among young adult workers in the offset color proof-printing department at a printing company in Japan [53]. They were chronically exposed to chlorinated organic solvents, including dichloromethane and 1,2-dichloropropane. Interestingly, IPNB or IPNB associated with invasive carcinoma was predominantly observed in the dilated intrahepatic and perihilar bile ducts, showing sclerosing cholangitis involving the peribiliary glands [54,55].

#### 2.1.3. Related Diseases Outside the Hepatobiliary System

(1) IPMN: Although approximately 10% to 40% of IPMNs are associated with extrahepatic malignancies, IPNB is rarely associated with IPMN synchronously or dyssynchronously in the same patient [48,56,57,58,59,60]. While both share some radiologic and histologic features, the significance of this coexistence and pathogenetical relations remain speculative. A 69-year-old woman developed invasive IPMN and underwent a pancreatectomy six months after hepatic resection of invasive IPNB. In that case, a molecular analysis revealed a *GNAS/KRAS* mutation in both the invasive IPMN and IPNB, suggesting that these two entities may share similar molecular alternations [56]. Alternatively, some could be an implantation of either or vice versa.

(2) Other diseases: IPNB and gastrointestinal stromal tumor and neurofibromatosis type 1 were found in a case of neurofibromatosis type 1 [61]. There have also been rare cases of IPNB in liver cirrhosis patients [62].

#### 2.1.4. Complication

(1). Hepatic gastric fistula and pancreatobiliary fistula: rarely, IPNB shows fistula formation to the adjacent organs, such as to the stomach and pancreas. In a previous case, laparotomy showed a markedly dilated B3 containing IPNB on the liver surface, which continued to the lesser curvature of the stomach, and IPNB was involved in hepatic gastric fistula [63]. Another case with co-occurrence of IPNB and IPMN also showed pancreatobiliary fistula [48].

(2). Seeding: A case of needle tract seeding of an intraductal papillary neoplasm of the bile duct (IPNB) after a percutaneous biopsy was reported. IPNB was seeded to the skin at the port site after a percutaneous needle biopsy [64].

### 2.2. Imaging and Endoscopy

#### 2.2.1. Cross Sectional Imaging

**Figure 1:**
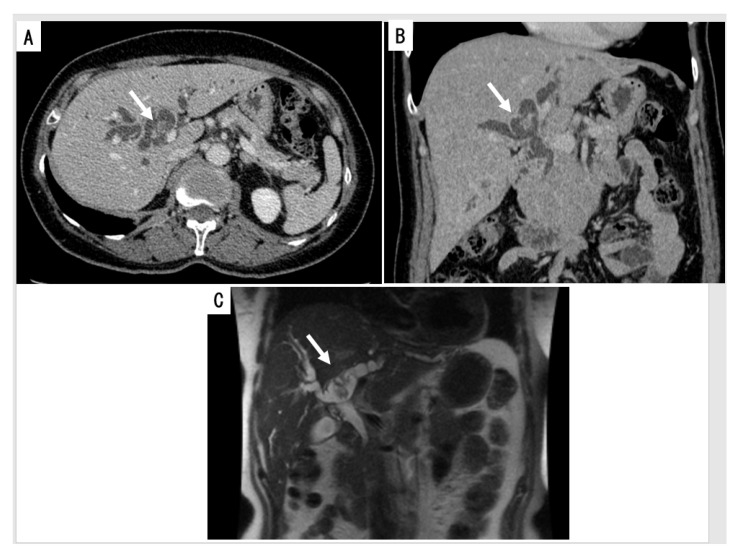
Enhanced computed tomography (CT) and magnetic resonance imaging (MRI) of intraductal papillary neoplasm of bile duct (IPNB). (**A**) Enhanced CT (horizontal section). The intrahepatic bile ducts are dilated with enhanced intraductal mass lesions (IPNB) (→). (**B**) Enhanced CT (coronal section). Mass lesions (IPNB) (→) are found in the dilated intrahepatic bile ducts. (**C**) MRI findings (T2 weighted image, coronal section). Bile duct reveals diffuse dilatation with low intensity tumors (IPNB) (→) at the hilar portion.

The most important morphological changes are the presence of (a) intraductal mass(es) and surrounding intraepithelial neoplastic biliary mucosa, (b) diffuse or segmental bile duct dilatation with or without cystic changes (maximum 126 mm) and (c) ductal and periductal invasion including macro-invasion of the liver [3,28,41,42,43,65,66,67] (Figure 1A,B). In ultrasound sonography (US), IPNB was recognizable by variable features, including hyperechoic nodules (37.5%), focal bile duct dilatation (37.5%) and diffuse bile duct dilatation with intraductal nodules (25%) [43]. A cystic mass may involve more than one lobe [66]. Magnetic resonance imaging (MRI) reveals IPNB as isointense to hypointense masses on T1-weighted images and hyperintense masses on T2-weighted images [65,66] (Figure 1C). Significant MRI findings for differentiating IPNB with an associated invasive carcinoma from non-invasive IPNB with intraepithelial neoplasia include an intraductal visible mass, tumor size ≥2.5 cm, multiplicity of the tumor, bile duct wall thickening and adjacent organ invasion [67]. MRI with magnetic resonance cholangiography (MRC) may be helpful for differentiating IPNB with an associated invasive carcinoma from non-invasive IPNB with intraepithelial neoplasia [67]. On computed tomography (CT), almost all cases in a previous report showed bile duct dilatation (98.2%) and an intraductal mass (92.9%) [43], and the enhancement pattern of IPNB is isodense or hyperdense during the late arterial phase and not hyperdense during the portal-venous and delayed phase. Other findings obtained by CT are infiltration of the neoplasm along the duct wall and intense rim enhancement at the base of the lesion.

#### 2.2.2. Cholangiography

**Figure 2:**
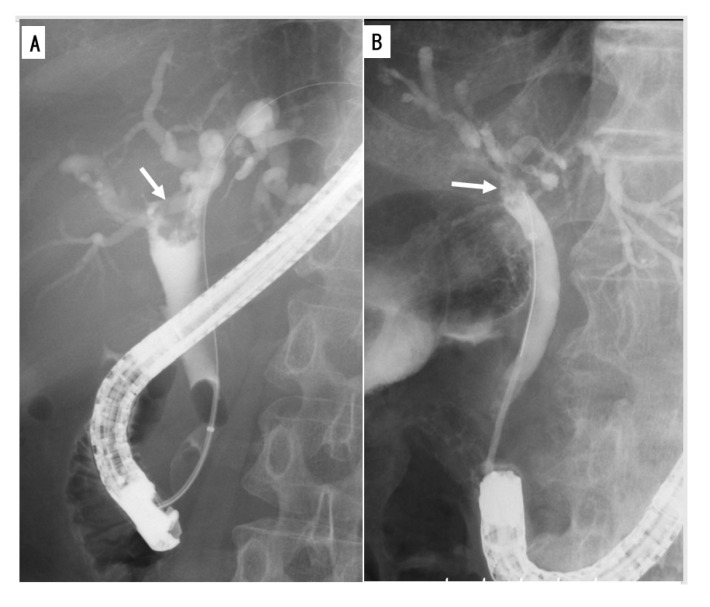
Endoscopic retrograde cholangiography of intraductal papillary neoplasm of bile duct (IPNB). (**A**) Balloon occluded cholangiography reveals dilated bile duct and filling defect (→) (IPNB) in the dilated bile duct at the hilar portion. (**B**) Cholangiography reveals filling defect (→) (IPNB) in non-dilated bile duct at the hilar portion.

Endoscopic retrograde cholangiography (ERC) and MRC are useful for depicting the entire bile duct in order to clarify the extent of IPNB [68,69,70]. ERC is useful for detecting mucobilia, which is seen in nearly one-third of patients with IPNB, as evidenced by diffuse dilatation of the bile duct with an irregular or amorphous filling defect (Figure 2A,B) [69,71]. Furthermore, in nearly half of patients, communication between the cyst and bile duct is demonstrated [15]. Brush cytological specimens and even tissue specimens are obtainable during ERC.

MRC is also a standard, noninvasive method for demonstrating the extent of narrowing or dilatation of the bile duct and multifocal intraductal tumors, but it cannot detect the presence of mucin overproduction in the bile duct [70,71]. IPNB usually shows a signal defect against bile juice, which presents with a high signal intensity. The affected bile duct in IPNB usually does not demonstrate stricture but sometimes demonstrates localized bile duct dilatation due to the mucin production of the tumor [46].

#### 2.2.3. Intraductal Ultrasonography (IDUS)

IDUS is reportedly useful for the evaluation of the lateral spread of CCA [72] and is a simple method for diagnosing the location of IPNB and assessing the depth and extent of invasion, even in the presence of thick mucin [68,73]. A forceps biopsy accompanied by IDUS can substantially improve the diagnosis rate of CCA [74,75].

#### 2.2.4. Cholangioscopy and Duodenoscopy

**Figure 3:**
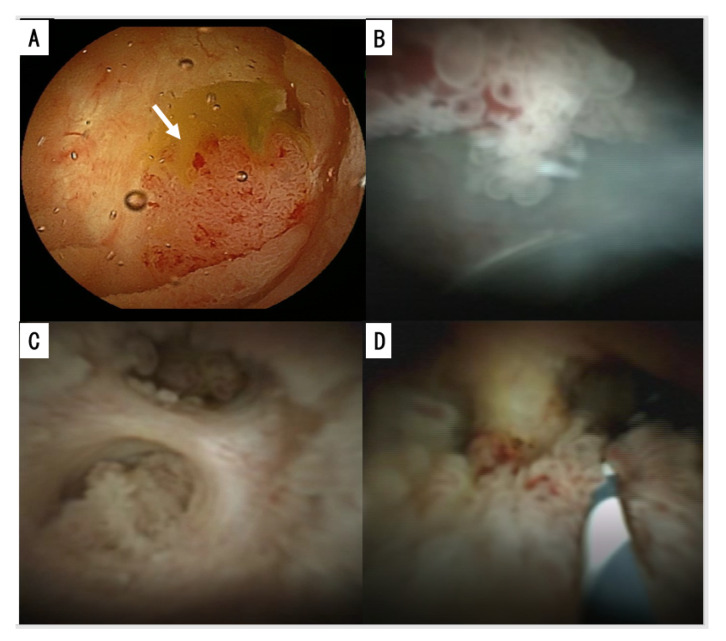
Per-oral cholangioscopic findings of intraductal papillary neoplasm of bile duct (IPNB) type 1 and 2. (**A**) Type 1 IPNB. Villous papillary tumor with mucin hypersecretion (→). (**B**) Type 1 IPNB. Fish egg like tumor with mucin hypersecretion in the bile duct. (**C**) Type 2 IPNB. Villous papillary tumor without mucin hypersecretion located in the bile duct at the hilar portion. (**D**) Type 2 IPNB. Fish egg-like tumor in the bile duct.

Peroral cholangioscopy (POCS) can visualize the bile duct directly and assess the extent of the tumor [75,76,77,78] (Figure 3A,B). POCS can be performed immediately after ERC with endoscopic sphincterotomy (EST) after the sufficient removal of mucin [79,80]. POCS can approach the bile duct directly and assess the surface and other characteristics of intraductal tumors and the surrounding biliary mucosa [81]. Characteristic findings of IPNB by cholangioscopy include papillary projections with or without the surrounding fish-egg-like or granular mucosa. In the observation of the fine mucosal structure, narrow-band imaging (NBI) is reportedly as good as or better than light imaging [75,76,77,78]. NBI reveals the fine mucosal structure and microvessels of the tumor. POCS allows for tissue and cytology samples to be obtained. Direct cholangioscopy should be considered as an adjunctive therapy to facilitate direct visualization and diagnostic sampling, especially in cases where advancement of the wire deep into the remnant bile duct is not feasible [82]. Furthermore, direct cholangiography with a biopsy was reported to facilitate determining the extent of type 1 IPNB and performing limited surgical resection [83].

Duodenoscopy frequently shows a dilated papillary orifice with mucin. However, the existence of the thick mucin filling the dilated biliary tree often prevents the visualization of intraductal tumors [68,84,85]. The luminal communication of IPNB with cystic changes with the adjacent bile duct can also be identified.

## 3. Pathologies of IPNBs

The gross pathologies and histologies of IPNB are dependent on the anatomical location of the tumor, tumor size, mucin hypersecretion, invasion, secondary bile duct changes, subtypes and structural and cellular atypia, as well as geographic factors [2,3,39,44,66,86].

### 3.1. Location along the Biliary Tree

IPNBs can develop in the large intrahepatic and extrahepatic bile duct but usually not in the intrahepatic small bile ducts [2,3]. The location of IPNBs along the biliary tree has varied widely among studies dependent on geographic variations [9,31,39,41,71]. The majority of IPNBs (67%) were located at the intrahepatic bile ducts in Asian countries [40,41], while in Western countries, they were more common in the extrahepatic bile ducts or hepatic hilum, and 24.2% were intrahepatic IPNB [9,15,23,35,47,63]. About 40% of IPNBs can present separate multiple lesions along the biliary tree, both synchronously and dyssynchronously [2,18,41,87,88]. Some may represent multiple occurrences of IPNB in the bile duct mucosa with a neoplastic predisposition, while others are due to intraluminal implantation or dissemination of neoplastic cells along the biliary tree. When IPNB exists in the intrahepatic bile ducts, it tends to be found in the left-sided biliary ductal system [35]. However, in *Opisthorchis viverrini* (OV)-infected patients, IPNB was found more commonly at the right than left intrahepatic ducts and had more peripheral than central locations [43]. Extrahepatic IPNBs show more aggressive pathologic features and a higher rate of invasion than intrahepatic IPNBs [25,30,40,41].

### 3.2. Gross Features

#### 3.2.1. Intraductal Tumors

**Figure 4:**
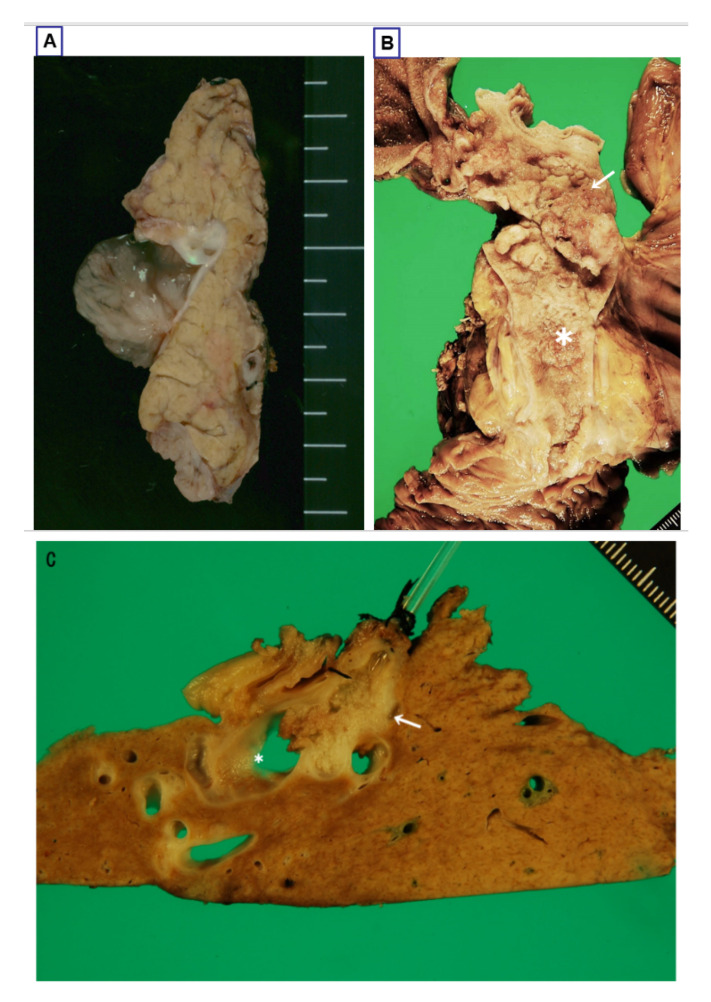
Gross features of intraductal papillary neoplasm of bile duct (IPNB). (**A**) Single papillary neoplasm in the extrahepatic bile duct is covered by visible much mucin layer (→). (**B**) Conglomerated polypoid lesions (→) and surrounding granular or rough mucosa (*) are regionally distributed in the perihilar and distal bile duct. (**C**) Papillary lesions in the cystically dilated intrahepatic bile ducts (*) are associated with invasion (→).

IPNBs present as single or isolated papillary or villous or polypoid exophytic growth (Figure 4A) or conglomerated and continuous papillary or villous or polypoid exophytic lesions (mixed smaller and higher) (Figure 4B). Some are limited to one part of the biliary tree, while others extend continuously for a considerable length and area over the bile duct mucosa [2,3,5,37]. Several cases have shown widespread extension [89]. According to the study by Kim et al. [37,90], 35% of cases were of polypoid appearance, 23% of cast-like, 28% of superficial spreading and the remaining 15% of cyst-forming type.

According to our recent study on the intraluminal external observation of 40 cases of IPNBs collected from Shizuoka Cancer Center, the IPNBs were grossly classifiable into three groups as follows: (a) an isolated, polypoid or papillary or villous lesion on the duct mucosa (Figure 4A); (b) conglomerated exophytic lesions (mixed low and high, and mixed papillary, villous, polypoid or large granular lesions) that distributed regionally or extended longitudinally in variable extent (Figure 4B); and (c) multiple (more than two) discrete and discontinuous exophytic nodules on the bile duct mucosa (Figure 4C). Some cases of group b appeared to involve multiple lesions, but histologically they were continuous neoplastic lesions. Group b was the most common (19 cases, 47.5%), followed by group a (13 cases, 42.5%) and group c (4 cases, 10%). Group b was further divided into relatively narrow-ranged lesions (12 cases) and wide-ranged lesions (7 cases). The latter may extend from the distal bile duct (intrapancreatic portion) to the bilateral intrahepatic bile ducts. However, there were no marked differences among these three gross patterns in the anatomical location, distribution of type 1 or 2 subclassification, four subtypes and stromal invasion (Table 2). Kim et al. also reported that the four gross types of IPNB based on surface observation showed no relation to the invasion tendency [37,90], suggesting that while these gross features may correlate with endoscopic findings of IPNBs, another approach is (or other approaches are) needed in order to correlate the gross features with other clinicopathological features, including stromal invasion and its depth and type 1 and 2 subclassifications.

The size of IPNBs, including cystic lesions, ranges from 0.5 to 16 cm (median: 2.2–6.0 cm) [37,66,91,92,93]. On the affected mucosa, the height of the main tumor from the adjacent biliary mucosa is at least 5 mm from the adjacent biliary mucosa in typical cases; however, some papillary neoplasms with a similar histopathology that are <5 mm but >3 mm in height are occasionally encountered [94]. IPNBs located in the intrahepatic bile ducts tend to be larger in both height and length than those in the extrahepatic bile ducts [2,25].

In addition to the main tumor, a variable proportion or extent of the mucosa around the main papillary lesions are rough and show visible granular or small papillary lesions continuous with the main lesion. The surrounding mucosal changes are continuous with the main tumor [2,87,95]. The internal surfaces of the cystic neoplastic lesions with mural papillary neoplasms are also rough or finely granular and micropapillary, suggesting that intraepithelial neoplastic lesions are continuous with papillary lesions. It is therefore plausible that IPNB is composed of (i) grossly visible main tumors and (ii) surrounding intraepithelial neoplasms.

#### 3.2.2. Mucin Hypersecretion

More than one-third of IPNBs (about 40%) show mucin hypersecretion, and the mucin layer covers the papillary lesions and fills the bile duct lumen [31,37,66,89,96]. Mucin hypersecretion is more frequently observed in intrahepatic IPNBs than in extrahepatic IPNBs [2,37]. Bile duct dilation is also more severe in mucin-hypersecreting IPNBs than in mucin-nonsecreting IPNBs. Ohtsuka et al. [31,96] also reported that mucin-hypersecreting IPNBs showed striking similarities to IPMN and were usually in situ carcinoma or minimally invasive, whereas IPNBs without mucin hypersecretion were frequently associated with invasive carcinoma. Mucin hypersecretion was significantly more frequent in patients with gastric and intestinal subtypes than in those with oncocytic or pancreatobiliary (PB) subtypes [97].

#### 3.2.3. Bile Duct Dilatation

Some IPNBs, particularly those arising in the extrahepatic bile ducts, are associated with cylindrical or fusiform dilatation of the bile ducts impacted by cast-like neoplasms, while other IPNBs, particularly those in the intrahepatic bile duct, present with marked macroscopic diffuse or segmental dilatation or unilocular or multilocular cystic dilatation with intraductal or intracystic mural neoplasms and mucus hypersecretion (Figure 4C) [3,37,41,49,66]. Such cystic IPNBs should be differentiated from MCN and other anomalous lesions, such as accessory gallbladder embedded in the liver parenchyma [19,20,98,99,100]. These cystic changes may involve one or even two hepatic lobes and represent cystic dilatation of the bile ducts, usually showing luminal communication with the adjacent bile duct, and are therefore not true cysts. Bile ducts with excessive mucin secretion located upstream and downstream from IPNBs are significantly dilated due to the large amount of mucin in the duct lumen.

#### 3.2.4. Classification Based on the Radio-Pathological Appearance

Several classifications have been proposed based on the gross and radiological appearance. Recently, Kim et al. [37] proposed a modified anatomical classification of IPNB: extrahepatic type, wherein the main lesions are confined to the extrahepatic hepatic duct; intrahepatic type, wherein the main lesions are located at the intrahepatic bile ducts; and diffuse type, wherein the main lesions are located over a wide are of the intrahepatic and extrahepatic bile ducts. Furthermore, those authors divided the intrahepatic type into two subgroups: the cystic form and duct-ectatic form. They reported that 265 (68.5%) of the 387 patients were intrahepatic, 103 (26.6%) were extrahepatic and 16 (4.1%) were diffuse type. Although intrahepatic IPNB showed a good long-term prognosis, relatively aggressive features were also found in the extrahepatic/diffuse type [101]. Similar to IPMN, there have been several reports of main duct-type and branch duct-type IPNB [102,103,104], although which part of the biliary system corresponds to the branch duct of the pancreas remains uncertain [38].

### 3.3. Histologies

#### 3.3.1. General Features

**Figure 5:**
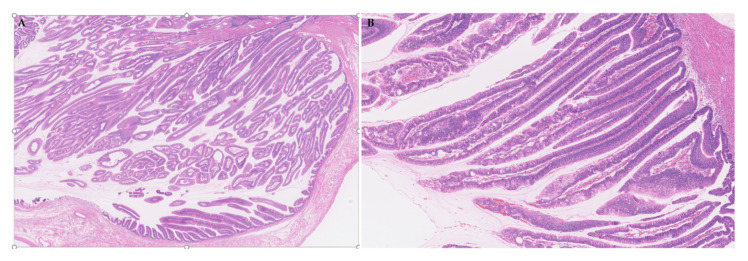
Histological features of intraductal papillary neoplasm of bile duct (IPNB). (**A**) In the dilated bile duct, papillary lesions with fine fibrovascular stalks and covered by lining epithelial (intestinal subtype) are seen. The surrounding mucosa adjacent to main tumor also shows micro-papillary-villous neoplastic lesions (H&E) in the figure legend should be changed to (×100, original magnification, H&E). (**B**) Villous neoplasm with fibrovascular stalks and lined by columnar epithelia in the distal bile duct resembles villous neoplasm of the colorectum (intestinal subtype) (H&E) in the figure legend should be changed to (×150, original magnification, H&E).

IPNBs are a preinvasive, papillary/villous biliary neoplasm with variable tubular components, covering fine fibrovascular stalks or with fibrous stroma in dilated bile ducts (Figure 5A,B). Some cases of IPNB, particularly oncocytic subtype, show mildly widened stroma due to edema and inflammatory cell infiltration [3]. The histology of IPNB is heterogeneous, depending on the subtypes, mucin production, grade of cytoarchitectural atypia, invasion and tumor location along the biliary tree [32,94].

#### 3.3.2. Four Subtypes

IPNBs are histologically classifiable into four subtypes based on their epithelial cell lineages: intestinal IPNB (iIPNB), gastric IPNB (gIPNB), pancreatobiliary IPNB (pbIPNB) and oncocytic IPNB (oIPNB) [2,3,32,37,97,105,106]. This subtyping is facilitated by immunohistochemistry to detect mucus core proteins and cytokeratins [37]. The main histologic and immunohistochemical features of IPNBs described in previous reports [2,30,97,106] are shown in Table 3. The intracellular mucin expression is dependent on the grade of epithelial atypia as well as the subtype. Regarding the incidence, iIPNB and pbIPNB are relatively frequent compared with gIPNB and oIPNB, and the incidence varies among reports and is dependent on geographical differences [15,37,44]. The presence of these four subtypes itself is considered a feature distinguishing IPNB from other biliary tumors and supports the notion that IPNB and IPMN share pathologic and phenotypic features [2,3,25,32,97].

While many IPNB cases are predominantly composed of an individual subtype, admixtures of foci of other subtypes and cases with controversial subtyping are sometimes observed. Intrahepatic IPNB tends to have an intestinal subtype, while extrahepatic type tends to have an intestinal or PB subtype [15]. There are no apparent differences in the predominant sex or age among the four subtypes of IPNB [9,15,35]. Furthermore, the gastric subtype is reportedly more commonly associated with low-grade dysplasia, while the PB subtype is usually associated with high-grade dysplasia and aggressive behavior [32,33,39].

Bile duct mucosa adjacent to or around the main tumor present intraepithelial micropapillary or flat neoplastic lesion around the main tumor. Grading and subtypes are always similar or identical to the main tumor, although the gastric epithelial neoplasm is also identifiable in the surrounding mucosa in oncocytic IPNB. This surrounding flat or micropapillary neoplastic lesion and main tumor make up the composite neoplasm of IPNB [2,87,95].

#### 3.3.3. Two Tiered Grading: Low- and High-Grade Dysplasia

Neoplastic epithelial cells display a spectrum of cytoarchitectural atypia ranging from none to borderline or even overt malignant changes, and invasive carcinoma can also be associated with IPNB [2,44,67,89]. A two-tiered grading system of low-grade dysplasia versus high-grade dysplasia, mainly based on these atypia, particularly nuclear changes, is applied to IPNB in order to delineate clinically significant examples from the insignificant ones [2,3,16]. High-grade IPNBs show hyperchromatic nuclei, nucleoli, nuclear and cellular pleomorphism and a loss of polarity, while low-grade IPNBs do not show these findings. Generally, about 10% to 40% of IPNBs are low-grade, while others are high-grade with or without low-grade foci (about 60% to 90%) [15,35,39,66,107,108,109,110]. Invasive carcinoma is frequently associated with high grade dysplasia.

#### 3.3.4. Invasion and Metastasis and Recurrence

##### Invasion

Stromal invasion is found at the base of intraductal tumors along with fibrovascular stalks near the base, and these affected fibrovascular stalks are usually widened. Stroma invasion can also develop at adjacent or surrounding intraepithelial neoplastic areas [95]. A surgical series demonstrated invasive carcinoma arising from IPNBs, with rates ranging from 31% to 74% [15,16,35,37,48,107]. The invasion is usually minimal when present in surgically resected IPNBs, probably because of the early detection of IPNBs due to biliary obstruction by the tumor or hypersecreted mucin [107]. About 62% of IPNBs were shown to be confined to the duct wall in previous studies, with the remaining 36% showing invasion through duct wall and invasion to adjacent organs [35,111]. However, in some geographical areas, most IPNB patients show invasion with frequent microinvasion of the liver [40,41].

The invasive parts of IPNBs usually show tubular adenocarcinoma with a desmoplastic reaction and only occasionally show foci of colloid carcinoma. The oncocytic subtype shows invasion of oncocytic adenocarcinoma.

Invasion is reportedly related to several factors. For example, invasion is more frequent in Western countries than in Asian countries [9,15,23,35,41,47]. Invasion also differs according to the anatomical location of IPNB, with approximately 30% to 50% of cases of intrahepatic IPNBs showing stromal invasion, whereas such invasion is more frequent in extrahepatic IPNBs (up to 90%), implying that intrahepatic IPNBs are less aggressive than extrahepatic IPNBs [15,30,37,94]. The depth (degree) of invasion is more progressed in extrahepatic IPNB than in intrahepatic IPNB. The frequency of invasive carcinoma in the pancreatobiliary subtype is significantly higher (72.7%) than in the gastric (26.7%) and intestinal (32.6%) subtypes [35,97]. As for type 1 and 2 subclassification, invasion was reported to be more frequent in type 2 than in type 1 [15]. As mentioned above, the gross features of IPNB were reported not to correlate with stromal invasion [37,90].

Kim et al. [37,97] reported that while IPNBs were classifiable into polypoid, cast-like, superficial-spreading and cyst-forming types, such gross features did not correlate with stromal invasion.

##### Lymph Node Metastasis

Lymph node metastasis is found in 6–8.2% of IPNBs at the time of surgical resection [15,17,37,41].

##### Recurrence

The recurrence rate reportedly ranges from 13–29% for all IPNB diagnoses and 47–62% among patients with invasive disease at the time of surgery [23,35,47,68,84]. The main recurrence sites are the liver, lymph nodes of the para-aortic area and hepatoduodenal ligament, bile duct (including anastomotic sites), proximal and distal bile ducts, abdominal wall, peritoneum and lung [15,111]. Interestingly, no significant differences in the incidences of recurrence sites have been reported between the type 1 and type 2 subclassification [15].

Local recurrence of IPNB or the new development of CCA after surgical resection of IPNB (3.7% of all IPNB patients, 5.6% of type 1 and 2.8% of type 2 patients [15,111]) may occasionally occur due to the implantation or cancerization of neoplastic cells [87,88]. While careful follow-up schedules for surveillance according to primary tumor location are needed after surgery [112], no significant differences in the rate of initial isolated locoregional recurrence or initial distant recurrence according to the tumor location have been reported.

### 3.4. Variants

#### 3.4.1. Bile Duct Dilatation with Microscopic IPNB (Superficial Spreading IPNB)

Some intraductal preinvasive neoplasms show diffuse dilatation of the bile ducts without visible intraductal tumors on imaging and macroscopic observation because of their microscopic size. Such patients underwent liver resection who presented with disproportionate dilatation of the bile duct with or without excessive mucin hypersecretion, without any visible mass or point of obstruction. For example, Nanashima et al. reported a case showing extensive bile duct dilatation filled with mucin and lined by a superficially spreading, microscopically identifiable, non-invasive biliary neoplasm despite no grossly visible identifiable papillary neoplasms [113]. Lim et al. also reported the imaging features of intrahepatic biliary intraductal papillary-mucinous neoplasm manifesting only as dilatation of the lobar or segmental bile ducts and spreading along the mucosa without forming a visible mass, noting that it was capable of producing a large amount of mucin [93]. Severe dilatation of the lobar or segmental intrahepatic bile ducts with crowding and severe atrophy of the hepatic parenchyma are a helpful imaging finding in such cases [114]. While these cases are usually non-invasive, some have shown microinvasion [40,41]. Several reports included such cases in IPNB and called them micropapillary IPNB or superficial spreading IPNB, and in one study, such cases accounted for 28% of all IPNBs [38,41,46,90,96]. However, they were not recognizable grossly, and the differentiation of such cases from micropapillary BilIN involving a considerable area of the bile duct mucosa remains controversial [4,5,6,7,14].

#### 3.4.2. IPNB Arising in Peribiliary Glands and Other Parts of the Liver

**Figure 6:**
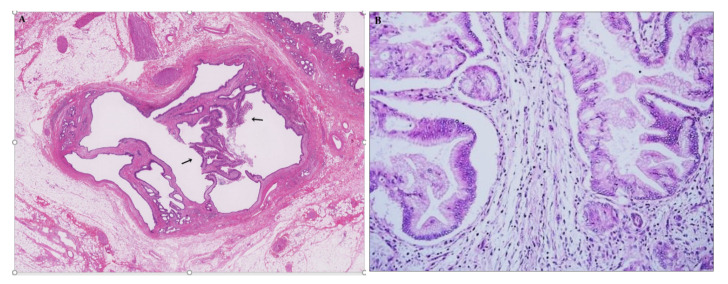
Cystic micropapillary neoplasm of bile duct. (**A**) The neoplastic lesion around the hilar bile duct shows cystic lesions with micropapillary epithelial growth (arrows). Lower magnification. H&E in the figure legend should be changed to (×50, original magnification, H&E). (**B**) Cystic micropapillary neoplasm shows pyloric gland changes and foveola appearance suggesting gastric phenotype. H&E in the figure legend should be changed to (×200, original magnification, H&E).

While a majority of IPNBs may arise from the epithelia lining the biliary tract [2,3], some cases of IPNB can derive from the peribiliary glands and then spread to the adjacent bile duct luminal mucosa [115,116,117]. A diverticulum-like appearance on imaging may be expected in such cases [91,113,116]. Recently, Pedica et al. reported that 4.6% of peribiliary cysts in alcoholic cirrhosis had low-grade IPNB confined to the peribiliary glands, suggesting that these lesions might be incidental and incipient IPNBs arising in the cystically dilated peribiliary glands. This finding underlined the possible role of the peribiliary glands in the development of IPNB [118]. Such cystic and micropapillary lesions affecting the peribiliary glands were also detected in 9 (1%) of 938 consecutive autopsy cases (Figure 6A,B) [117]. The hyperplastic epithelium of these lesions is variably positive for gastric-type mucins, such as MUC5AC and MUC6, resembling pancreatic intraductal papillary mucinous neoplasm of the branch duct type [118,119], and the degree of atypia ranges from low- to high-grade. A single case in the original report was associated with invasive CCA [120]. This type of cystic and micropapillary lesion may be a counterpart of branch duct IPMN, as the peribiliary glands and their conduits are branching ducts from the main bile duct [121,122].

## 4. A Novel Subclassification of IPNB Based on Cytoarchitectural Alterations

Recently, Umemura et al. [108] reported that more than half of IPNBs (64%) were diagnosed as in situ carcinoma and the remaining are IPNB with invasive carcinoma and, interestingly, no cases of low-grade dysplasia were found in their series. Several other studies also reported that all intraductal papillary neoplasms with or without invasion are carcinoma [109,110]. The diagnostic criteria for low- and high-grade dysplasia of IPNB may not be the same among global regions, institutions and pathologists, and sampling error may also be a challenging issue for this two-tiered system, particularly in small specimens from IPNB, a grossly visible tumor with non-homogeneous histologies. In this context, the application of this two-tiered grading system thus seems to be not enough, and an additional, alternative approach may be needed for the categorization of IPNBs based on their cytological alterations and structural changes of the IPNB as a whole.

Recently, Japan–Korea expert pathologists discussed the possibility of subclassification of IPNB based on the structural changes of IPNB as a whole combined with a two-tiered grading system (low-grade and high-grade-dysplasia), and proposed type 1 and type 2 subclassification [33,94,111].

### 4.1. Morphological Features Characterizing Type 1 and 2

#### 4.1.1. Type 1

**Figure 7:**
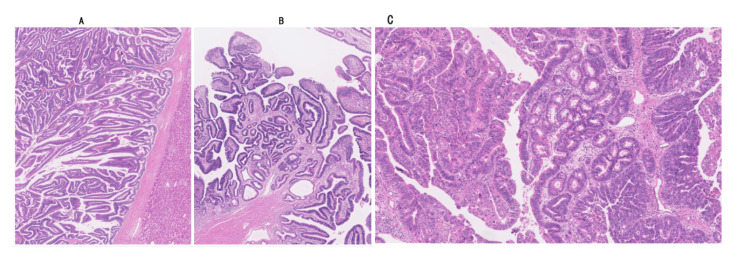
Histologies of type 1 and type 2 IPNB. (**A**) Type 1 IPNB. Regular growth, mainly villous pattern, is recognizable. Fibrovascular stalks are thin. H&E in the figure legend should be changed to (×100, original magnification, H&E). (**B**) Type 2 IPNB. Irregular growth pattern showing papillary and tubular patterns with focal widened fibrovascular stalk. H&E in the figure legend should be changed to (×100, original magnification, H&E). (**C**) Type 2 IPNB. Complicated structures such as densely compact tubular, cribriform, solid and papillary growth patterns are recognizable. H&E in the figure legend should be changed to (×150, original magnification, H&E).

This type of IPNB shows regular papillary, villous or tubular structures and a relatively homogeneous appearance. Papillary fibrovascular stalks are generally thin (depending on the subtype), while fibrovascular stalks are variably widened at the basal side in some cases. The structures are regular and more or less homogeneous in appearance (Figure 7A). IPNBs with low-grade dysplasia (about 10% of all IPNBs) and those with high grade dysplasia with regular structures (30%) belong to type 1.

#### 4.1.2. Type 2

This type shows irregular structures and a non-homogeneous appearance and is composed of high-grade dysplasia and irregular structures (60% of all IPNBs) (Figure 7B). In addition, this type commonly shows foci of complicated lesions or structures, such as cribriform, compact tubular and solid components or patterns, as well as relatively large cystic changes within the tumor and foci of bizarre cells and nuclear changes appearing as overt malignancy (Figure 7C). Coagulative necrosis is also experienced in type 2. Neuroendocrine differentiation has been reported in type 2 IPNB [123]. These complicated features are easily identifiable and reproducible lesions and recommended to be applied to this subclassification in practical diagnosis. Interobserver interpretation and consensus on the regularities and homogeneity also characterizing this subclassification may facilitate more usage of this subclassification.

Previously, Albores-Saavedra et al. described invasive and non-invasive well-differentiated papillary cholangiocarcinoma as a morphological variant of extrahepatic bile duct carcinoma [124,125,126]. There are also similar reports of papillary carcinoma of the extrahepatic bile duct and intrahepatic bile duct [38,109,110,124,125,126,127,128,129]. The morphologies and description of these carcinomas may be regarded as similar or identical to IPNB confined to the ductal lumen and wall or with minimal invasion, and they are considered to be IPNBs, specifically type 2, in the proposal by the Japan–Korea Pathologist group and in the WHO classification [3,94].

Taken together, previous studies and discussions have suggested that type 1 IPNBs are associated with a non-invasive phenotype, intestinal and oncocytic subtypes, frequent development in the intrahepatic bile duct and excessive mucin production. In contrast, type 2 IPNBs are associated with an invasive phenotype, intestinal and PB subtypes and frequent development within the extrahepatic bile duct. These pathological characteristics are summarized in Table 4.

### 4.2. Characteristic Findings of Types 1 and 2 in Recent Clinical Studies

According to recent clinical studies using many IPNB cases and this subclassification [15,33,108,111,130], types 1 and 2 were found to show similar but also different clinicolaboratory and pathologic features. Interestingly, these studies reproduced the above-mentioned proposed characteristics features of type 1 and 2 IPNB [3,94]. The main features of type 1 and 2 IPNBs reported by recent clinical studies are shown in Table 5. For example, mucobilia was frequent in type 1 in comparison with type 2. Interestingly, long term post-operative outcome was significantly favorable in type 1 compared with type 2.

Thus far, no radiological approaches have been developed for distinguishing type 1 and 2 IPNB.

## 5. Genetic Changes of IPNBs

### 5.1. General Survey

At present, there are no genetic alterations that have been established as common across all IPNB cases. Several genetic studies have assessed the alterations on one or more genes in IPNBs, but the genes mutated and their frequency vary among analyses due to the small number patients examined [9,34,38,56,131,132,133,134,135]. Studies of *GNAS* have shown a marked difference in the frequencies of *GNAS* codon 201 mutations, ranging from low (2–2.9%) to higher rates of mutation (29–50%) [9,34,56,131,132,133,135], potentially due to population ethnicity as well as geographical differences and subcategories associated with special risks in IPNB [35].

Recently, by next-generation sequencing (NGS), Yang et al. [130] and Aoki et al. [111] identified frequent mutations in IPNBs (Table 6). Mutations of several genes, such as *KRAS, TP53, GNAS* and *CTNNB1*, were found to be relatively frequent in IPNBs in both series.

Herein, these genetic changes of IPNBs are discussed with respect to four subtypes, type 1 and 2 subclassification, and similarities to IPMN.

### 5.2. Four Subtypes

As in IPMN, four subtypes of IPNBs show characteristic histologies and several different clinicopathological behaviors [35,111], which may be related to genetic changes unique to individual subtypes.

#### 5.2.1. IPNB with Intestinal Differentiation

Intestinal IPNB belonging to type 1: Among recurrent mutations in IPNBs, Thsai et al. reported that, in East Asia, *GNAS* mutations were detected in fewer than half of all cases of IPNB, and all cases with *GNAS* mutations had intestinal differentiation [132,133]. Mutations in *RNF43*, a tumor suppressor gene, and *KRAS* mutation, in addition to *GNAS* mutation, were also shown to be frequent in intestinal IPNBs [132,133]. Recent report showed that when divided into intrahepatic and extrahepatic locations, intestinal IPNBs arising in the intrahepatic bile ducts and belonging to type 1 frequently present with *GNAS*, *KRAS* and *RNF43* mutations [132,135], suggesting that intestinal IPNBs, particularly those arising in the intrahepatic bile duct, show similar mutations as in IPMN [111,132,133,135]. This group could be a distinctive category of IPNB different from other IPNB categories as Yang et al. suggested [130].

Intestinal IPNB belonging to type 2: Intestinal IPNB arising in the extrahepatic bile duct and belonging to type 2 did not harbor *GNAS* mutations but did show mutations in *SMAD4*, *PIK3CA*, *APC* and *CTNNB1* [135], suggesting the different genetic changes from IPMN [136] and the above-mentioned intestinal IPNB belonging to type 1.Yang et al. also reported such intestinal IPNB belonging to type 2 with mucin hypersecretion and positive MUC2 different from intestinal IPNB with *GNAS* mutation and also from *CTNNB1* mutated non-intestinal IPNB (see below) as an another category of IPNB [130].

#### 5.2.2. IPNB with Non-Intestinal Differentiation

Fujikura et al. reported that mutations in *APC* or *CTNNB1*, both of which belong to the Wnt/β-catenin pathway, were observed in 43% of 14 cases of non-intestinal IPNB (5 gastric, 6 PB and 3 oncocytic subtypes) [137]. *GNAS* mutations were absent in their non-intestinal series. APC and β-catenin are part of the same oncogenic pathway, so alterations in an activation of β-catenin or inactivation of *APC* are sufficient to fully activate the WNT/β-catenin pathway. APC and CTNNB1 and the subsequent activation of the WNT/β-catenin signaling pathway could be unique for IPNBs with the non-intestinal subtypes. There is another report that the pancreatobiliary subtype arising in the extrahepatic bile ducts also harbors a *CTNNB1* mutation [130]. Indeed, IPNBs with *CTNNB1* mutations were of the PB subtype, frequently located in the extrahepatic bile duct, and lacked mutations in *KRAS*, *APC*, *RNF43* and *GNAS* [130,132,133]. Such IPNBs, therefore, appear genetically different from their pancreatic counterpart, as mutations of *APC* and *CTNNB1* are not or are only rarely observed in IPMN [135]. Given these previous findings, the activation of the Wnt/β-catenin signaling pathway associated with *APC* and *CTNBB1* mutation may be involved in the development and progression of non-intestinal-type IPNBs, particularly the pancreatobiliary subtype [130,137], and the genetic alterations of this type differ from those seen with IPMN [130,136]. So far, genetic changes unique to gastric subtype of IPNB remain to be clarified.

#### 5.2.3. IPNB with Oncocytic Differentiation

A recent study detected frequent and recurrent fusion genes in both oncocytic subtypes of both IPNB and IPMN [138]. Singi et al. [138] detected PRKACA or PRKACB-related fusion genes in all 23 oncocytic tumors investigated (20 IPMNs and 3 IPNBs), and these fusion events were not present in other pancreatobiliary tumors, including 23 CCAs and 16 IPMNs of other subtypes, demonstrating the specificity of this molecular event in oncocytic subtype of both IPMN and IPNB. Another recent study revealed that onocytic IPNB and oncocytic IPMN showed different expression patterns in several signal pathways, as well as an increased expression of follistatin (FST) and reduced apoptotic activity compared with other subtypes of IPNB and IPMN [139]. These finding suggest that the unique molecular signaling in oncocytic IPNB and oncocytic IPMN differs from other subtypes, which may facilitate the separation of oncocytic IPMN from other subtype of IPMN [140,141].

### 5.3. Type 1 and 2 Subclassification

While type 1 and 2 IPNBs share many features, they also present different clinicopathological features and molecular alterations. For example, type 1 presents favorable post-operative outcomes in comparison with type 2, and type 1 shares many features with IPMN but type 2 is variably different from IPMN [2,32,39,94].

Recently, mutations in genes of IPNBs were compared between type 1 and 2 lesions [111,130,135]. Aoki et al. reported that among mutations of genes, mutations in *KRAS* were significantly more frequent in type 1 IPNBs than in type 2 [111], and mutations in *GNAS* and *RNF43* were only found in type 1 IPNBs. These mutations are also reported to be frequent in IPMNs [136]. In this context, type 1 IPNBs share many features with IPMNs [94]. In contrast, type 2 IPNBs were reported to show frequent mutations of *TP53*, *SMAD4* and *KMT2C* mutations and aberrant expression of TP53 and SMAD4 but rarely harbored *GNAS* mutations [111] (Table 7). Yang et al. also reported that *TP53* mutations were common in type 2 IPNBs [130]. These genetic studies suggest that IPNBs consist of at least two distinct types of pathogenesis from the perspective of gene mutations, and the type 1 and 2 subclassifications may reflect these genetic subcategorizations.

### 5.4. Similarities and Dissimilarities to IPMN

IPNB is viewed as the biliary counterpart of IPMN, though recent studies showed that IPNBs, particularly type 1, and IPMN share many clinicopathological features but type 2 were variably different from the prototypes of IPMN [2,15,21,24,33]. So far, the similarities or dissimilarities in genetic mutations between IPNBs and IPMNs have not been fully investigated [34,111,133]. Some studies have suggested that certain genetic changes may be shared by IPNB and IPMN, but there are many differences in the oncogenic pathways leading to the development of intraductal papillary tumors in these two regions [34,130,135]. Intestinal IPNBs subclassified into type 1 were associated with *KRAS*, *GNAS* and *RNF43* mutations which are reportedly common in IPMNs [132,136], indicating that type 1 IPNB was a biliary counterpart of IPMN [111,130,132,133,135]. Mutations in *APC* or *CTNNB1*, both of which belong to the Wnt/β-catenin pathway, were observed in non-intestinal IPNBs, particularly pancreatobiliary subtype, but these mutations are not or are only rarely observed in IPMN [130,136,137], thus these IPNB with activation of the Wnt/*β-catenin* signaling pathway may not be a biliary counterpart of IPMN [130,136]. Instead, oncocytic IPNB and oncocytic IPMN present the same genetic and molecular process [138,139], thus they could be a counterpart to each other. Since oncocytic IPMN has been separately classified as intraductal oncocytic papillary neoplasm (IOPN) from IPMN [140,141], oncocytic IPNB may be considered independent from other IPNB subtypes.

Taken together, the differences of genetic changes of IPNB in several categories as above mentioned, indicate that IPNB could be a heterogenous disease, and approaches to individual subtypes or subcategories are needed in future studies on IPNB.

## 6. Molecular Alterations and Signal Pathways in Development and Progression of IPNBs

Molecular alterations and signal pathways cloud be evaluated in several ways in IPNB. First, according to the different backgrounds and risks, more than one altered signal pathways and molecular changes may be involved in an individual lesion’s pathogenesis. Second, IPNBs may undergo several pathologic steps in the progressions reflected in the molecular and signaling deregulation.

### 6.1. Different Backgrounds and Risks

Chronic biliary inflammation, including hepatolithiasis and liver fluke infection, may induce the production of reactive oxygen or nitrogen species, leading to DNA damage and neoplastic changes of the biliary epithelia followed by the development of IPNB [142,143,144,145,146,147,148]. Pathogenesis and progression of IPNB could be different in several types of infections or suspected carcinogens.

For example, IPNBs with liver fluke infection, particularly *Clonorchiasis sinensi* (CS), tended to have a more pancreatobiliary phenotype (MUC1+/MUC2-/CDX2-) [142,143], whereas IPNBs negative for CS were characterized by the intestinal phenotype (MUC2+/CK20+) [143]. In CCA associated with *Opisthorchis viverrini (OS)* infection, mutation of cancer-related genes *TP53* (mutated in 44.4% of cases), *KRAS* (16.7%), *SMAD4* (16.7%), *RNF43* (9.3%) and *GNAS* (9.3%) were reported and they may be involved in deactivation of histone modifiers, activation of G protein signaling and loss of genome stability [145].

In IPNBs with exposure to chlorinated organic solvents including 1,2-dichloropropane and/or dichloromethane, γ-H2AX, a marker of DNA double strand break, was significantly increased in foci of IPNB and invasive carcinoma. These organic solvents might act as a carcinogen for biliary epithelial cells by causing DNA damage, hypermethylation, many somatic mutations and C:G-to-T:A transitions with substantial strand bias as well as unique trinucleotide mutational changes of GpCpY to GpTpY and NpCpY to NpTpY or NpApY, thereby contributing to carcinoma development [146]. In this series, carcinoma cells expressed programmed death-ligand 1 (PD-L1) in all cases of CCA derived from IPNB were frequently associated with PD-L1-positive mononuclear cells, PD-1-positive lymphocytes and CD8-positive lymphocytes infiltrating within the tumor, suggesting that the PD-1/PD-L1 axis was aberrantly activated and favorable response to immune checkpoint inhibitor therapy could be promising [147,148].

### 6.2. Low- and High-Grade Dysplasia

IPNBs may undergo sequentially progression from low-grade to high-grade and then to invasive adenocarcinoma [2,3,9,35]. In parallel with this progression, IPNBs have shown the stepwise acquisition of molecular alterations affecting common oncogenic pathways, such as cell-cycle-related molecules [9,144,149,150,151,152]. While the genetic mutations significantly associated with high-grade IPNB in the reports using NGS remain controversial [111,130], there are several interesting studies. For example, high-grade IPNBs were reported to show an increased expression of cyclin D1 [134,151,153]. The p53 expression showed a stepwise accumulation with increasing tumor grade, suggesting that it may play a role in the later stage of disease [111,144,151]. A decreased membranous expression of β-catenin and E-cadherin is an early event in the tumorigenesis and grading of IPNB [153]. Cyclin D1 and c-myc were frequently positive in the IPNB, and interestingly, nuclear β-catenin accumulation was observed in the IPNB, indicating aberrations of Wnt signaling in the tumorigenesis of the IPNB [144,152]. This aberration may be activated preferentially in non-intestinal IPNBs by using a whole exome sequencing study [137]. p16 aberrations occur early in low-grade IPNB and precede the aberrant expression of p53 [9,149]. High-grade IPNBs were reported to show an increased expression of Ki-67, mCEA and CA19-9 [134]. The increased expression of autophagy-related proteins in IPNB in hepatolithiasis suggests the role of dysregulated autophagy at an early stage of IPNB development [150].

HepPar I was frequently expressed in non-invasive IPNB, particularly non-oncocytic IPNB, but not in invasive IPNB. The overexpression of the polycomb group protein enhancer of zeste homolog 2 (EZH2), a family of proteins responsible for cellular differentiation, is also involved in the progression of IPNB [149,150,152] and may be associated with malignant behavior in IPNB in parallel with the upregulation of MUC1 expression and downregulation of MUC6 expression [150,152].

Schlitter et al. reported that mutated RAS was already identifiable even in low grade dypsplasia of IPNB [9]. KRAS mutation may result in the constitutive activation of MAPK pathway [111,130]. The rate of *KRAS* mutations was significantly also higher in high-grade IPNBs, and *KRAS* mutations were significantly associated with the tumor size and Ki-67 expression [134].

### 6.3. Stromal Invasion and Occurrence of Complicated Lesions

Stromal invasion and complicated lesions such as solid or cribriform pattern and foci of bizarre cells and nuclear changes appearing overt malignancy reflecting more aggressive behaviors are commonly found in type 2 but not in type 1 IPNBs [2,32,33,94]. The expression of MUC1 was significantly more frequent in invasive cases (87.5%) than in non-invasive IPNBs (50%) [149,151], suggesting carcinogenesis leading to invasive tubular adenocarcinoma is associated with increasing aberrant expression of MUC1. Interestingly, IPNB leading to colloid carcinoma is associated with MUC1-negativity [142,144], suggesting the involvement of different molecules in these two invasive processes in IPNB. Aoki et al. reported that the MUC1 expression was immunohistochemically observed more frequently in type 2 (100%) than in type 1 (59%) (Table 7) [111]. The aberrant expression of other cancer-related molecules such as p53 and SMAD4 was also more frequent in type 2 (64.3% and 42.9%) than in type 1 (9.1 and 9.1%) [111,114,130], supported by frequent mutations in *PT53*, *PICK3CA* and *SMAD4* in type 2 IPNB than type 1 (Table 8) [111,130,135]. Schlitter et al. also reported loss of SMAD in the late phase of IPNB [9]. The deregulated signal pathways related to these genetic changes may be involved in stromal invasion and also occurrence of complicated lesions in IPNBs. Interestingly, *KRAS*, *RNF43* and *GNAS* mutation were more frequent in type 1 than type 2, reflecting that these mutations are more importantly related genetic changes of IPNB with respect to the tumorigenesis maintaining similarities to IPMN and/or lower aggressive characters of IPNB.

### 6.4. Targettable Genes and Proteins in IPNB

Taken together, the genes mutated and proteins aberrantly expressed in type 2 IPNB may be involved in deregulated signal pathways responsible for stromal invasion and occurrence of complicated lesions resulting in aggressive behaviors of IPNB. Thus, these genes/proteins and resultant deregulated signal pathways could be possible targets by specific therapeutic challenges including drugs against IPNB. In addition, further analyses in the molecular mechanisms common in all IPNBs may also lead to discovery of targets for drug therapy.

## 7. The Diagnosis, Treatment and Prognosis, Including the Post-Operative Outcomes, of IPNBs

A high potential for high-grade dysplasia (or carcinoma in situ) and frequently invasive nature but usually confined to the duct [33] at the diagnosis are hallmarks of IPNB. Furthermore, the recurrence rate of IPNB is high, being found in up to 29% of cases, potentially impairing the long-term outcomes [17].

### 7.1. Preoperative Diagnosis

**Figure 8:**
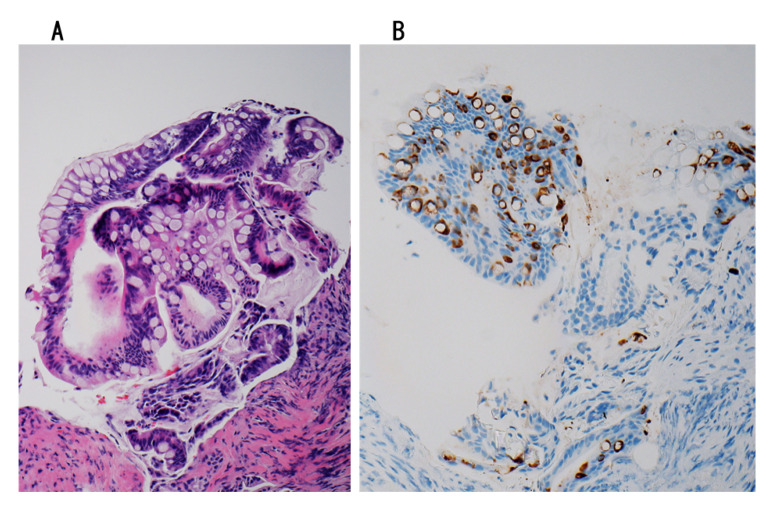
Endoscopic biopsy of intraductal papillary neoplasm of bile duct (IPNB). (**A**) Papillary neoplasm of the bile duct present intestinal differentiation interspersed with goblet cells and low-grade dysplasia. Type 1 is strongly suspected. H&E in the figure legend should be changed to (×200, original magnification, H&E). (**B**) Goblet cells interspersed in the papillary lesions in the bile duct are positive for MUC2. Immunostaining of MUC2. H&E in the figure legend should be changed to (×200, original magnification, H&E).

The diagnosis of IPNB can be challenging due to its varying clinicoradiological presentations [17,37]. Imaging plays a major role in not only the diagnosis of IPNB but also the management strategy employed, and with improvements in imaging equipment and diagnostic technology, including cholangioscopy, the early diagnosis rate of IPNB is increasing [8,28,33,35,67]. CT and MRI are frequently used in the diagnosis of IPNB, with typical findings being biliary tract dilatation and an intraductal mass. A preoperative tissue diagnosis provides important information, particularly when a villous or papillary neoplasm is obtained (Figure 8A,B). However, its practical application remains limited at present. A preoperative misdiagnosis of IPNB can occur in clinical practice due to its low incidence, lack of specific tumor markers and unclear pathogenesis [18,154].

### 7.2. Treatment

All patients with IPNB should be considered for treatment because high-grade dysplasia with invasion is frequently seen in IPNB, and papillary tumors and associated mucin often cause recurrent cholangitis and obstructive jaundice, even if the tumors exhibit low-grade dysplasia [35,41,97,155].

#### 7.2.1. Surgical Resection

Early surgical resection is strongly advisable for radiologically suspected IPNB to prevent disease progression [15,39], and surgery is performed in the same manner as surgical resection for conventional p/dCCA and large duct iCCA [15,18,23,31,33,82,96,97,155,156]. Regional lymphadenectomy should also be performed.

Extrahepatic IPNBs tend to be removed by bile duct resection or pancreato-duodenectomy [37,83], while IPNBs of the intrahepatic bile duct and perihilar bile ducts tend to be removed by hepatobiliary resection [37]. Local excision of the biliary tract is applicable for lesions of the middle part of the extrahepatic bile duct, and pancreato-duodenectomy is suitable for distal bile duct tumor [18,83]. In cases of IPNB with low- to high-grade dysplasia and limited superficial spread, limited resection preserving the organ function can be selected [45,83,96,150]. In such cases, a precise preoperative diagnosis is essential. In contrast, in cases of IPNB with extensive superficial spread that may have positive margins, even after extensive resection, resection for the whole biliary tree by liver transplantation with or without pancreatico-duodenectomy is theoretically regarded as the only curative treatment [82,96,157]. However, the efficacy of this procedure remains unclear, and the indication of liver transplantation for patients with IPNB is very limited at present [156].

The type 1 and 2 subclassification of IPNB may be helpful for making decisions concerning the surgical approach, as type 1 IPNB usually shows less aggressive behavior than type 2 IPNB and develop preferentially in the intrahepatic bile duct [15,94,105,111]. Therefore, a significant difference in the surgical procedures used has been found between these two types [15]. Hepatic resection is mainly performed for patients with type 1 IPNB, whereas patients with type 2 IPNB undergo hepatic resection, pancreato-duodenectomy or bile duct resection.

Since a better long-term prognosis can be achieved in IPNB patients by ensuring sufficient surgical resection, it is important to accurately localize the main lesions and the surrounding intraepithelial neoplastic area and establish a proper extent of resection based on the preoperative radiologic imaging findings and a pathological evaluation of biopsy specimens, just as with surgery for conventional CCA [37,76,77,78,79,83,84,118]. R0 resection was reportedly achieved in 90% of IPNB patients [35]. Aggressive further resection should be considered when the resection margin is involved with any residual lesion, including dysplasia in IPNB [37].

#### 7.2.2. Non-Surgical Treatment

When major surgery is not possible, some palliative treatments, such as percutaneous transhepatic biliary drainage, and cholangioscopic electrocoagulation and adjuvant therapies, including chemotherapy, iridium-192 intraluminal therapy and argon plasma coagulation, have been reported [156,157,158,159]. Partial hepatectomy followed by palliative chemotherapy has also been reported [160]. Recently, the treatment of IPNB using argon plasma coagulation with a follow-up period of more than two years was newly reported [161].

### 7.3. Post-Operative Outcomes and Influencing Factors

The median postoperative survival of IPNB patients is favorable compared with that of conventional CCA [5,18,35,162,163]. The rates of lymph node metastasis or distant metastasis are much lower in IPNB than conventional CCA [18,27,96,154]. For example, Gordon-Weeks et al. evaluated a total of 476 specimens of IPNBs, including those associated with invasion, and the survival rate after resection was 96% at 1 year, 79% at 3 years and 65% at 5 years [35]. Lee et al. reported that the 1-, 3- and 5-year recurrence-free survival (RFS) rates for surgically resected IPNB were 93.8%, 79.1% and 70.0%, respectively [67].

Many factors have been reported to be associated with worse or favorable outcomes after surgical resection of IPNB (Table 9) [24,44,88,108,126,163,164], although most factors for IPNB are either not well established or still controversial, aside from lymph node metastasis and type 1 and 2 subclassification [15,111].

The main prognostic factors are discussed below.

#### 7.3.1. Gross Features

Morphologic classifications, including the modified anatomical classification proposed by Kim et al., were shown to have no effect on the survival [37]. However, Luvira et al. reported that cystic IPNB and micropapillary IPNB (disproportional bile duct dilatation in the absence of any discernible tumor) showed a favorable post-operative prognosis, while IPNB with bilateral dilatation of intrahepatic bile ducts had a poor prognosis [41].

#### 7.3.2. Anatomical Location

Matsumoto et al. considered that patients with intrahepatic IPNBs had more favorable pathological characteristics and postoperative survival outcomes than those with extrahepatic IPNBs [162]. The independent prognostic factor for the RFS was shown to be the tumor location, as extrahepatic IPNB had a poorer 5-year RFS than intrahepatic IPNB (51.7% vs. 91.4%) [118]. However, there have been several reports that an IPNB being located in the extrahepatic or intrahepatic bile duct had no relation to the postoperative survival rate [37,165].

#### 7.3.3. Invasion

The degree of invasion, including the UICC stage, is a poor prognostic factor [35,107], but a multivariate analysis failed to show this significance [39]. Recently, Lee et al. reported that the RFS rates were significantly lower in patients with significant MRI findings of IPNB with an associated invasive carcinoma, as previously mentioned, than in those without significant MRI findings [67]. Significant MRI findings of IPNB with an associated invasive carcinoma have a negative impact on the RFS [67]. However, the data remain controversial.

#### 7.3.4. Subtypes

There have been several reports that the histologic subtype has no effect on the survival [37,39]. A previous study found no significant difference in the post-operative survival between cases of PB and intestinal subtypes [35,166]. Kubota et al. demonstrated no significant association between the cumulative survival rates and four subtypes [15]. In contrast, Kim et al. reported that patients with the PB subtype had a significantly worse survival than those with the gastric or intestinal subtype [97]. Aoki et al. noted that the 5-year survival rate was 10% in IPNB of the gastric, intestinal and oncocytic subtype but was 57.9% in cases of the PB subtype [111]. The MUC6 expression in the tumor showed only a marginal influence on the predicted prognosis [107]. Given these previous findings, the data remain controversial.

#### 7.3.5. Subclassification: Type 1 or 2

Several recent reports, including Kubota’s multi-institutional study, have shown that type 1 is associated with a favorable prognosis, while type 2 is associated with a poor prognosis [15,32,111]. The 1-, 3-, 5- and 10-year cumulative survival rates (CSRs) for Type 1 IPNB were 96.1%, 85.2%, 75.2% and 58.5%, respectively, while those for Type 2 IPNB were 94.6%, 69.1%, 50.9% and 26.8%, respectively (*p* < 0.001) [15]. The average disease-specific survival rate was 90.9% in type 1 patients and 58.7% in type 2 patients (*p* < 0.001) [111].

#### 7.3.6. Surgical Margin

Previous multivariate analyses have shown that the bile duct margin status with carcinoma in situ is an independent prognostic factor reflecting a poor prognosis [37,107]. The tumor recurrence rate and proportion of locoregional recurrence were found to be significantly greater among patients with in situ carcinoma than among those with negative bile duct margins, including patients with low-grade dysplasia [107]. Surveillance after resection of IPNB is critical in patients with a residual extrahepatic bile duct, even in those with negative resection margins [82,107].

At the bile duct margin, Kubota et al. showed that there were no significant differences in the CSR or CDFSR between groups with positive and negative bile duct margins for type 1 as well as type 2 [15]. This indicates that the condition of the bile duct margin is not associated with the prognosis of IPNB, regardless of type 1 or 2 disease. The presence of invasive components in the surgical margin is associated with a poor prognosis [28,37]. However, local recurrence along the biliary tree is occasional [15,111]. IPNB with superficial mucosal spreading or a diffuse type [37] tends to have a positive resection margin.

#### 7.3.7. Metastasis

Lymph node metastasis has been shown to be an independent poor prognostic factor [15,37,38,41,111,130]. IPNB patients with lymph node involvement are at an increased risk of tumor recurrence. [111].

#### 7.3.8. Others

Multiplicity of IPNB along the biliary tree, bilateral intrahepatic and extrahepatic dilatation and the degree of perineural invasion and expression of CK20 in the tumor are reported as post-operative poor prognostic factors [15,35,37,41,112,114].

### 7.4. Staging (TNM)

The staging of CCA derived from IPNB follows the TNM classification for intrahepatic, perihilar and distal CCA [167].

## 8. Conclusions

IPNB is a rapidly emerging, newly recognized pre-invasive neoplasm of the bile duct with high malignant potential and is frequently followed by invasive CCA. Grossly, IPNBs are characterized by predominantly intraluminal growing epithelial neoplasm(s) with fine fibrovascular stalks. The affected bile ducts show dilatation due to intraductal tumor mass and mucus hypersecretion, and they are clinically recognizable by imagings and endoscopy. IPNBs are classifiable into four subtypes by their epithelial cell lineages: intestinal subtype is the most common followed by gastric, pancreatobiliary and oncocytic subtypes. Almost all cases of IPNB are graded into high-grade by a two-tiered grading system. To supplement cytoarchitectural grading, a novel subclassification of IPNB into types 1 and 2 is recently proposed: type 1 is composed of low-grade IPNB and high-grade IPNB with regular structures, and type 2 is composed of high-grade IPNB with irregular structures and constantly shows complicated lesions. Type 1 and 2 IPNBs share several clinicopathological features but also present different characters. Particularly, long-term post-operative survival is significantly favorable in type 1 in comparison with type 2. Genetically, IPNBs showing mutations in *KRAS*, *GNAS* and *RNF43* belong to type 1, particularly the intestinal subtype, while IPNBs showing mutations in *CTNNB1 and APC* with activation of the Wnt/*β-catenin* signaling pathway and lacking mutations in *KRAS*, *GNAS* and *RNF43* belong to the pancreatobiliary subtype. IPNB showing mutation of *TP53*, *SMAD4* and *PIK3CA* might reflect occurrence of aggressive histological features including stromal invasion associated with type 2. Similarities to pancreatic IPMN are found in the intestinal subtype belonging to type 1 and oncocytic subtype. Further comprehensive analyses of molecular alterations and genetic changes concerning the four subtypes, type 1 and 2 subclassifications, staging and anatomical locations along the biliary tree are mandatory and may lead to discovery of novel therapeutical target. Recognition of this pre-invasive neoplasm will encourage a better understanding of clinicopathological features and pathogenesis of CCA as well as therapeutic challenging against CCA at the pre-invasive and early invasive stages.

## Figures and Tables

**Table 1:** Proposed, accepted and unrecommended terms for intraductal papillary neoplasm of bile duct (IPNB) by the World Health Organization (WHO) Classification of Tumours (2019) [1].

WHO Proposed Term	WHO Accepted Terms		WHO Unrecommended Terms
IPNB (intraductal papillary neoplasm of bile duct)	Biliary papilloma and papillomatosis		Biliary adenoma
			Intestinal adenoma
			Papillary (villous) adenoma
			Tubulopapillary (tubule-villous) adenoma
			Non-invasive papillary neoplasm (carcinoma)
			Papillary carcinoma
			Mucin-secreting biliary tumor

**Table 2:** Characteristics of gross features of intraductal papillary neoplasm of bile duct (IPNB).

Clinicopathological Features	Localized Papillary Type	Conglomerated Type		Multifocal Type
		Narrow Ranged	Wide Ranged	
Number of cases	17	19		4
		12	7	
Intra/Extra/Both	4/13/0	8/8/3		0/4/0
		8/3/1	0/5/2	
Type 1: Type 2	2:15	5:14		1:3
		3:9	2:5	
I/G/O/PB	10/1/1/5	9/4/3/3		2/2/0/0
		4/3/2/3	5/1/1/0	
Stromal invasion	4	13		2
		8	5	

Intra, intrahepatic bile duct; Extra, extrahepatic bile duct; both, intrahepatic and extrahepatic bile duct; I, intestinal subtype; G, gastric subtype; O, oncocytic subtype; PB, pancreastobiliary subtype.

**Table 3:** Characteristics of four subtypes of intraductal papillary neoplasm of bile duct (IPNB).

Four Subtypes	Definitions	Immunohistochemistry
Intestinal subtype	* Neoplastic epithelia lining the fibrovascular cores showing columnar cells with pseudostratified, cigar-shaped nuclei and basophilic or amphophilic cytoplasm and with variable amounts of supranculear mucin, resembling colorectal neoplasms. * Presenting mainly villous structures, papillovillous or mixed papillotubular or tubular patterns reminiscent of tubular or villotubular neoplasms of the colorectum.	* Positive for CK20 and/or * CDX2 in their cytoplasm * Positive for MUC2 in goblet cells
Gastric subtype	* Neoplastic lining composed of tall columnar cells with basally oriented nuclei and abundant pale mucinous cytoplasm, reminiscent of the gastric foveolar epithelium, intermingling with glandular areas reminiscent of gastric pyloric glands. * High-grade dysplasia showing columnar epithelia with more complicated structures including irregular papillary or tubular or microcystic changes with atypical features.	* Positive for MUC5AC in the foveolar areas and for MUC6 in the pyloric gland portions.
Pancreatobiliary subtype	* Ramifying fine and thin branches and papillae covered by cuboidal to low columnar epithelia with acidophilic or amphophilic or pale cytoplasm, and by a less mucinous appearance * Round, hyperchromatic nuclei, prominent nucleoli * Including the cases with irregular papillary architecture with more stratified nuclei and solid or comedo-like structures with atypical structures and cells and nuclei.	* Positive for S100P and MUC1 and negative for MUC5AC.
Onocytic subtype	* Complex and arborizing papillae with delicate fibrotic and edematous stroma, lined by one to several stratified layers of cuboidal to columnar cells with abundant eosinophilic granular cytoplasm and occasional hyaline globules * Hyperchromatic, round, large, and fairly uniform nuclei * Frequent secondary intraepithelial lumina.	* Positive for MUC5AC.

CK, cytokeratin; MUC, mucus core protein. *, main features of each subtype are itemized.

**Table 4:** Characteristic pathologic features of type 1 and 2 intraductal papillary neoplasms of bile duct (IPNB).

Pathologic	Features	Type 1 IPNB	Type 2 IPNB
Structures		Regular villous, papillary or	Irregular and complicated villous,
		tubular structures	papillary or tubular structures
		Homogeneous appearance	Heterogeneous appearance
Grade of neoplasm intraepithelial		Low-grade dysplasia	High-grade dysplasia with no or
		High-grade dysplasia with	minimal foci of low-grade dysplasia
		foci of low-grade dysplasia	High-grade dysplasia
Location at the biliary tree		Usually intrahepatic bile duct	Intrahepatic and extrahepatic bile duct
Mucin overproduction		Frequent	Infrequent
Stromal invasion		Infrequent	Common
Subtypes		Intestinal and oncocytic subtype	Pancreatobiliary and intestinal subtype
Similarities to prototypic subtypes of IPMN		Similar (depending on subtype)	Different variably (depending on subtype)
Complicated lesions such as solid or cribriform pattern, coagulative necrosis, cystic changes		Almost absent	Frequent
Bizarre cellular and nuclear changes		Absent	Infrequent
Fibrovascular stalks		Thin to slightly widened (depending on subtype)	Thin to widened (depending on subtype)

**Table 5:** Main features of type 1 and type 2 intraductal papillary neoplasm of bile duct (IPNB) based on recent four published papers, cited from references [15,32,33,39,108,111,130].

Clinicolaboratory Features	Type 1	Type 2
Prevalence in IPNB	30–75%	25–70%
Clinical features		
* Age range	65–67 years	69–72 years
* Sex	Slightly male predominant	Slightly male predominant
* Jaundice, fever, abdominal pain	20%, 10%, 17%	39%, 18% 24%
* Background: hepatolithiasis	11%	6%
cholecystolithiasis	16%	8%
choledocholithiasis	9%	4%
* Elevations of AST, ALT, ALP,	relatively lower	relatively higher
γ-GTP, and T. Bililubin	relatively lower	relatively higher
* Level of CEA and CA19-9		
Gross features		
* Location:		
intrahepatic	58–68%	14–27%
hilar, extrahepatic	32–35%	48–64%
mixed	7%	22%
* Tumor size	2–205 mm	2–220 mm
* Communication between		
cyst and bile duct	45%	50%
* Mucobilia	29–86%	12–21%
Histological features		
* Four subtypes (I:G:PB:O)	18–48%:23–32%:12–23.5%:6–32%	0–39%:51–86%:6.7–1.4%%:0–3%
* Similar to prototypic IPMN	Similar	Variably different
* Low-: high-grade dysplasia	4.5–7.9%:32–68%	0–0.6%:5.8–29%
* Stromal invasion	27–50%	71–94%
Lymph node metastasis	0.5–5.8%	21.4–14.7%
Post-operative outcome		
* 5 year cumulative survival rate	75.20%	50.90%
* 5 year cumulative disease- free	64.10%	35.30%
year		

*, subcategories of each factor; AST, aspartate transaminofearase; ALT, alanine transamirase; ALP, alkaline phosphatase; γ-GTP, γ-glutamyl transferase; CEA, carcinoembryonic antigen; CA19-9, carbohydrate antigen 19-9; I, intestinal subtype; G, gastric subtype; PB, pancreatobiliary subtype; O, oncocytic subtype; IPMN, intraductal papillary mucinous tumor (pancreas).

**Table 6:** A list of recurrent mutations in intraductal papillary neoplasm of bile duct (IPNB) examined by next-generation sequencing, cited from references.

Yang et al. (Taiwanese, 37 Cases) [130]	Aoki et al. (Japanese, 35 Cases) [111]
KRAS (49%)	TP53 (34.3%)
GNAS (32%)	KRAS (24%)
RNF (24%)	STK11 (25.7%)
APC (24%)	CTNNB1 (17.1%)
TP53 (24%)	APC (14.3%)
CTNNB1 (11%)	SMAD4 (14.3%)
	GNAS (11.4%)
	PBRM1 (11.4%)
	ELF3 (8.6%)
	KMT2C (8.6%)
	NF1 (8.6%)
	PIK3CA (8.6%)
	ARID1A (5.7%)
	ARID2 (5.7%)
	BAP1 (5.7%)
	BRAF (5.7%)
	EPHA6 (5.7%)
	ERBB2 (5.7%)
	KMT2D (5.7%)
	RNF43 (5.7%)

%, percentage of positive cases.

**Table 7:** Altered expression of cancer related protein in intraductal papillary neoplasm of bile duct (IPNB), cited from references [111].

Cancer Related Protein	Type 1 (22 Cases)	Type 2 (14 Cases)
MUC1 *	11	14
P53 *	2	9
SMAD4	2	6

These cancer related molecules are relatively frequently expressed in type 2 in comparison with type 1; *, statistically significant.

**Table 8:** Frequency of mutations in type 1 and type 2 intraductal papillary neoplasm of bile duct (IPNB), cited from references [111,130].

Mutated Genes	Type 1 (21 Cases)	Type 2 (14 Cases)
KRAS *	10	1
GNAS *	4	0
RNF43	2	0
TP53 *	3	9
SMAD4 *	0	5
ARID1A	0	2
ERBB2	0	2

Upper half shows mutations relatively frequent in type 1, while lower half shows mutation of genes relatively frequent in type 2. *, statistically significant.

**Table 9:** A list of factors related to post-operative prognosis of intraductal papillary neoplasm of bile duct (IPNB).

Factors			Worse Prognosis
Clinical features			Lymph node metastasis, older age, jaundice, elevation of serum CA19-9 and CEA
Pathological factors of tumor			Multiplicity, perineural invasion, pancreatobiliary subtype, mucin
			hypersecretion, low and high grade dysplasia, tumor expression of CK20
			in tumor tissue, MUC1 expression in tumor,
Location			Extrahepatic location
Subtypes			Pancreatobiliary subtype
Subclassification			Type 2
Staging			Stromal invasion
			UICC staging, periductal invasion
Surgical margin			R1, R1/R2, ductal margin with high grade dysplasia (‘carcinoma in situ’),
			ductal margin with low-grade dysplasia
			Favorable prognosis
Pathologic factors of tumor			Cystic IPNB with micropapillary lesion, intrahepatic location, no invasion,
			low-grade dysplasia, MUC6 expression in tumor tissue
Subclassification			Type 1
Surgical margin			Negative surgical margin
